# Effectiveness of two intensive treatment methods for smoking cessation and relapse prevention in patients with coronary heart disease: study protocol and baseline description

**DOI:** 10.1186/1471-2261-12-33

**Published:** 2012-05-15

**Authors:** Nadine Berndt, Catherine Bolman, Lilian Lechner, Aart Mudde, Freek WA Verheugt, Hein de Vries

**Affiliations:** 1 Department of Psychology, Open University of the Netherlands, Heerlen, The Netherlands; 2 Department of Cardiology, Onze Lieve Vrouwe Gasthuis, Amsterdam, The Netherlands; 3 Department of Health Promotion, Faculty of Health, Medicine and Life Sciences, and School for Public Health and Primary Care (CAPHRI), Maastricht University, Maastricht, The Netherlands

**Keywords:** Coronary heart disease, Smoking cessation, Face-to-face counselling, Telephone counselling, Nicotine replacement therapy, Cost-effectiveness study

## Abstract

**Background:**

There is no more effective intervention for secondary prevention of coronary heart disease than smoking cessation. Yet, evidence about the (cost-)effectiveness of smoking cessation treatment methods for cardiac inpatients that also suit nursing practice is scarce. This protocol describes the design of a study on the (cost-)effectiveness of two intensive smoking cessation interventions for hospitalised cardiac patients as well as first results on the inclusion rates and the characteristics of the study population.

**Methods/design:**

An experimental study design is used in eight cardiac wards of hospitals throughout the Netherlands to assess the (cost-)effectiveness of two intensive smoking cessation counselling methods both combined with nicotine replacement therapy. Randomization is conducted at the ward level (cross-over). Baseline and follow-up measurements after six and 12 months are obtained. Upon admission to the cardiac ward, nurses assess patients’ smoking behaviour, ensure a quit advice and subsequently refer patients for either telephone counselling or face-to-face counselling. The counselling interventions have a comparable structure and content but differ in provider and delivery method, and in duration. Both counselling interventions are compared with a control group receiving no additional treatment beyond the usual care. Between December 2009 and June 2011, 245 cardiac patients who smoked prior to hospitalisation were included in the usual care group, 223 in the telephone counselling group and 157 in the face-to-face counselling group. Patients are predominantly male and have a mean age of 57 years. Acute coronary syndrome is the most frequently reported admission diagnosis. The ultimate goal of the study is to assess the effects of the interventions on smoking abstinence and their cost-effectiveness. Telephone counselling is expected to be more (cost-)effective in highly motivated patients and patients with high SES, whereas face-to-face counselling is expected to be more (cost-)effective in less motivated patients and patients with low SES.

**Discussion:**

This study examines two intensive smoking cessation interventions for cardiac patients using a multi-centre trial with eight cardiac wards. Although not all eligible patients could be included and the distribution of patients is skewed in the different groups, the results will be able to provide valuable insight into effects and costs of counselling interventions varying in delivery mode and intensity, also concerning subgroups.

**Trial registration:**

Dutch Trial Register NTR2144

## Background

Cardiovascular disease is the leading cause of death and the second largest source of healthcare costs in Western Countries [1]. A third of cardiovascular disease mortality and hospital admissions are caused by coronary heart disease [2,3]. Smoking cessation after a first coronary event such as a myocardial infarction significantly reduces the risk of mortality, hospitalisation rates and reoccurrence [4-6]. Smoking cessation is thus very relevant to patients with established coronary heart disease. Nevertheless, over half of those patients who were smokers prior to hospitalisation for coronary heart disease continue smoking after hospital discharge [7,8]. Cardiac patients who continue smoking after hospitalisation are characterised as highly dependent smokers with no or low future intentions to quit [9]. Earlier research showed that providing brief cessation support is not effective enough to help cardiac patients to quit smoking permanently [10,11]. Hence, there is a need for more intensive smoking cessation interventions for this patient group. Interventions proven to increase smoking cessation rates in hospitalised smokers in general are promising [11,12]. Compared with the benefits of prevention, and reduced morbidity and mortality, the healthcare costs of these interventions are low [13-19].

In the Netherlands, effective interventions for smoking cardiac patients which also suit nursing practice are scarce. A cardiac minimal intervention strategy (CMIS) meant for ward nurses to help smoking patients to quit was implemented on a large scale in Dutch cardiac wards [20]. Despite the moderate effect of the CMIS on smoking behaviour [10], its public health impact was hindered because of implementation difficulties in practice [21,22]. Only 28% of all Dutch cardiac ward nurses consistently and adequately used the CMIS [23]. Nurses reported barriers to its adequate implementation in hospital units as the difficulty of providing aftercare, lack of time and other priorities than smoking cessation support [22-27]. Because of the lack of intensive, and feasible interventions, many Dutch patients have continued smoking, resulting in high risks of (re)current coronary events or mortal closure.

Intensive smoking cessation interventions are, in general, recommended to cover the need for new (cost-)effective interventions for smoking cardiac patients [11,28]. Evidence suggests that interventions combining personalised behavioural counselling with pharmacological treatment are most likely to increase quitting rates in hospitalised (cardiac) patients [11,29-33]. It is recommended that this treatment begins during hospital admission [34] and continues for more than a month after hospital discharge with numerous contact moments [29,35]. Preferably, these interventions include quit advice by cardiologists [36], and a counselling approach integrating relapse prevention and motivational interviewing strategies [37-39]. Effectiveness can further be increased by intervening with regard to specific patient characteristics associated with a higher risk of continued smoking after hospitalisation for a cardiac event, such as symptoms of depression, anxiety and a Type D (distressed) personality [40-45].

Nicotine Replacement Therapy (NRT) has been shown to increase quitting rates, either delivered singly [31] or in combination with behavioural counselling [12,46-49]. The nicotine delivered by NRT has similar or less severe effects than nicotine obtained through cigarette smoking and for cardiac patients it has proved to be safer than smoking [50,51]. NRT provision is recommended for hospitalised smokers as it aids patients to suppress withdrawal symptoms [31], irrespective of their intention to quit [11,34,52].

Reviews of telephone counselling (TC) and face-to-face counselling (FC), both intensive smoking cessation behavioural interventions, concluded that they were equally effective in enhancing quit rates in general populations [11,32,33]. The significance of TC for cardiac inpatients is indicated by the fact that it includes multiple telephone sessions during a follow-up period of several months [29,53]. A study by Reid et al. (2006) reported an increase in smoking abstinence rates in cardiac patients who received TC in addition to usual care consisting of bedside counselling and NRT use in-hospital [54]. An earlier study revealed the efficacy of a nurse-based programme for cardiac inpatients motivated to quit, including bedside counselling and six follow-up calls in the four months after discharge. The cessation rate in the experimental group was twice as high as in the usual care group [55]. The significance of FC for the general population of smokers has been reported [32], though its effectiveness for cardiac patients is less clear. A meta-analysis showed that psycho-educational programmes for cardiac patients were effective, revealing a significant difference in smoking cessation rates between intensive and less-intensive interventions [56].

TC is less intensive than FC and hence might be more convenient for certain smokers [57]. There are strong indications that patients with high quit motivation profit more from TC than those with low quit motivation [33]. Moreover, low socio-economic status (SES) groups, often with low quit motivation, may profit more from FC than higher SES groups [58], although they are recognised as less successful quitters [59]. Previous studies indicated that low SES groups have higher coronary heart disease rates and smoking prevalence than high SES groups [60,61], and are less likely to quit smoking successfully while generally revealing lower intentions to quit [62,63].

The study described here started in 2009. It examines within the cardiac inpatient setting the (cost-)effectiveness and feasibility of TC and FC combined with NRT. The studies described above provided the rationale for making such interventions the subject of this evaluation study. TC and FC incorporate the same elements but differ in delivery mode and intensity. This paper describes the aims, hypotheses and design of the study, the intervention components of TC and FC, and the inclusion rates of the study participants and their baseline characteristics. The effectiveness study specifically compares health outcomes and smoking cessation outcomes, whereas the cost-effectiveness study also compares these outcomes with the costs of the interventions, including usual care.

On the basis of previous studies we expect to find 55% quitters at 12 months in both intervention groups against 35% in the control group [10,29,35,64]. Since reviews concluded that both interventions (TC and FC) were equally effective in general populations [32,33,65], we expect to find significant differences in smoking abstinence between usual care on the one hand and experimental conditions on the other. We furthermore expect to find differential effects within the experimental conditions: it is hypothesised that low SES groups profit more from FC compared with higher SES groups. It is also hypothesised that FC is more (cost-)effective for patients with low quit motivations, whereas TC is more (cost-)effective for patients with high quit motivations and a higher SES.

## Methods/design

### Setting

Forty-six cardiac wards of urban, leading clinical and academic hospitals throughout the Netherlands were approached for potential participation. Thirteen of these wards that did not offer any form of smoking cessation intervention to cardiac patients (unlike care as usual) were recruited. Five of these wards were still rejected by the research team because of incomparability with the other wards in terms of their constitution (i.e. they had a combined heart and lung unit). The remaining eight cardiac wards were accepted for the study.

In line with the Dutch Medical Research Ethics Committee (MERC) rules [66] the study protocol was submitted for approval to the MERC of the VU medical center Amsterdam. After approval by this MERC (MEC 2009/215; NL27637.029.09), the MERCs and/or Board of Directors of each hospital endorsed execution of the study. The study was registered with the Dutch Trial Registration (NTR2144).

### Design

An experimental study using cross-over randomization at the ward level with a baseline measurement at hospital admission and post-measurements at six and 12 months after baseline was conducted. All cardiac wards first provided care as usual and subsequently the two experimental conditions one after the other. Table 1 shows a schematic representation of the serially implemented conditions. After completion of care as usual, four of the cardiac wards started implementing TC, and after its completion they implemented FC. The other four cardiac wards implemented the experimental conditions in reverse order. After each condition there was a one month period without inclusion of any patient.

**Table 1:** Outline of the study design

**Cardiac wards 1 to 4:**	**Cardiac wards 5 to 8:**
O_1_ UC O_2_ O_3_	O_1_ UC O_2_ O_3_
O_1_ FC O_2_ O_3_	O_1_ TC O_2_ O_3_
O_1_ TC O_2_ O_3_	O_1_ FC O_2_ O_3_

O_1_: baseline measurement with questionnaire; *UC* Usual Care condition; *FC* Face-to-face Counselling experimental condition; *TC* Telephone Counselling experimental condition; O_2_: six months follow-up measurement by telephone interview; O_3_: 12 months follow-up measurement by telephone interview and biomedical measurements (saliva, cholesterol, blood pressure).

### Sample size calculations

Power and sample size calculations were conducted for the main outcome, seven-day point prevalence abstinence (PPA) after 12 months. Power calculations (one-sided; *p* < 0.05, Power = 0.80) showed that 193 patients per condition were needed to detect significant differences in PPA and possible interaction effects of the interventions with motivation to quit and SES. Advanced statistical methods, however, allowed adjustment for potential baseline confounding variables and improved the statistical power. Therefore, the required sample size was reduced by 30% [67], resulting in the need for N = 135 in each condition. Assuming 15% attrition at the 12-month follow up, 155 patients per condition needed to be recruited, totalling 465 patients for the three conditions.

### Baseline procedure

After approval was received from the Board of Directors and Ethical Review Committees of each hospital, the face-to-face counsellors (16 in total, i.e. two nurses from each of the cardiac wards that participated in the study) received training to provide FC. Ward nurses and cardiologists received written and oral instructions of the study with a written stepwise protocol and information sheet on the inclusion process of eligible patients, a flowchart with instructions and addresses for referral. Over an 18-month period beginning in December 2009, the nurses asked all patients admitted to the cardiac ward if they had smoked in the month prior to admission. If the answer was yes, the nurses reviewed the information sheet with the inclusion criteria, approached medically stable patients at the bedside, provided the necessary information about the study to patients, and invited eligible smoking patients to take part in the study. If patients agreed to take part, the nurses asked them to sign an informed consent form. Subsequently, at hospital admission, nurses administered the questionnaire to patients and registered relevant patient data on a separate form. Nurses also produced a summary of patients’ prescribed medication from their case history.

### Participants: inclusion and characteristics

Inclusion criteria for patients were: smoking on average ≥5 cigarettes per day in the month prior to admission or, if not smoking, having quit smoking less than four weeks before admission; being ≥18 years of age; being admitted to the cardiac ward for less than 96 h and being hospitalised because of a coronary heart disease (acute coronary syndrome, stable angina, and other forms of chronic and acute heart diseases) following the standards of the ICD-10 [68]. Exclusion criteria were language limitations that would impede completion of the self-administered questionnaire, a medically unstable cardiac situation or cognitive impairments.

**Figure 1:**
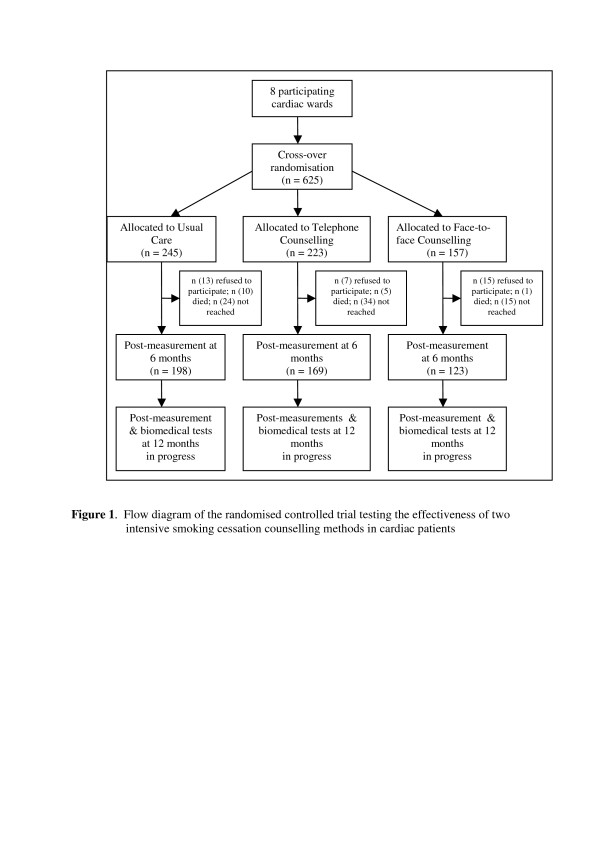
Flow diagram of the experimental study testing the effectiveness of two intensive smoking cessation treatment methods in cardiac patients.

As shown in Figure 1, 245 cardiac patients were included in the usual care condition, 223 in the experimental condition of TC, and 157 cardiac patients in the experimental condition of FC. With inclusion rates varying from 42 to 121 patients per cardiac ward, rates varied considerably per cardiac ward (see Table 2).

**Table 2:** Differences in inclusion rates per cardiac ward (N = 625)

	**Total**	**UC group**	**TC group**	**FC group**
	**N (%)**	**N (%)**	**N (%)**	**N (%)**
Cardiac ward 1	42 (6.7)	21 (8.6)	20 (9.0)	1 (0.6)
Cardiac ward 2	97 (15.5)	32 (13.1)	40 (17.9)	25 (15.9)
Cardiac ward 3	121 (19.4)	50 (20.4)	38 (17.0)	33 (21.0)
Cardiac ward 4	91 (14.6)	32 (13.1)	31 (13.9)	28 (17.8)
Cardiac ward 5	115 (18.4)	39 (15.9)	36 (16.1)	40 (25.5)
Cardiac ward 6	52 (8.3)	21 (8.6)	23 (10.3)	8 (5.1)
Cardiac ward 7	64 (10.2)	30 (12.2)	25 (11.2)	9 (5.7)
Cardiac ward 8	43 (6.9)	20 (8.2)	10 (4.5)	13 (8.3)

*Note.* Cardiac ward 1 is located in an academic university hospital. All other cardiac wards are located in leading clinical hospitals.

As presented in Table 3, patients were predominantly male with a mean age of 57 years. Education levels were relatively equally distributed over low, intermediate and high education. Most of the patients had an admission diagnosis of acute coronary syndrome (ACS) or had been diagnosed with ACS previously and were admitted to the hospital for treatment with a percutaneous coronary intervention (PCI) or a coronary artery bypass graft (CABG). During the six months prior to admission a few patients had been admitted to the hospital owing to a cardiac event. Nicotine dependence was moderate, and patients reported smoking 21 cigarettes on average per day before hospital admission. Although the majority of the patients had not made any quit attempt over the past 12 months, one-third reported on admission that they had not smoked over the past seven days. There were no significant differences between the three groups in terms of demographic characteristics. Nicotine dependence was significantly higher, however, in the control group than in the experimental groups.

**Table 3:** Baseline characteristics of the total sample and comparability of the three groups

	**Total (N = 625)**	**UC (n = 245)**	**TC (n = 223)**	**FC (n = 157)**
Variables	N (%)/M (SD)	N (%)/M (SD)	N (%)/M (SD)	N (%)/M (SD)
Gender (male)	457 (73.1%)	183 (74.7%)	163 (73.1%)	111 (70.7%)
Age	55.8 (11.08)	55.8 (11.88)	55.31 (10.53)	56.5 (10.57)
Marital status				
Married with/without children	413 (67.8%)	160 (67.5%)	151 (68.5%)	102 (66.7%)
Single/divorced/widow	196 (32.2%)	77 (32.5%)	68 (31.5%)	31 (33.3%)
Education level ^a^				
Low education	244 (40.5%)	97 (41.8%)	83 (38.1%)	64 (41.8%)
Intermediate education	233 (38.6%)	83 (35.8%)	88 (40.4%)	62 (40.5%)
High education	126 (20.9%)	52 (22.4%)	47 (21.6%)	27 (17.6%)
Disease diagnosis and/or treatment ^b^				
ACS (unstable angina, non-stemi, stemi)	530 (84.8%)	210 (85.7%)	191 (85.7%)	129 (82.2%)
Stable angina	53 (8.5%)	16 (6.5%)	23 (10.3%)	14 (8.9%)
Other diagnosis/unknown	42 (6.7%)	19 (7.8%)	9 (4.0%)	14 (8.9%)
Previous hospital admission (yes)	122 (20.2%)	48 (20.7%)	37 (17.0%)	37 (24.0%)
Nicotine dependence ^c^	5.25 (2.18)	4.99 (2.28)*	5.27 (2.14)	5.62 (2.01)
Average cigarettes per day	21.13 (13.04)	19.69 (10.64)	21.83 (15.78)	22.31 (11.82)
Seven-day abstinence at admission (PPA) (yes)	183 (29.3%)	79 (34.3%)	64 (29.4%)	40 (27.6%)
Quit attempts over the past 12 months (yes)	188 (30.5%)	87 (36.3%)	55 (24.9%)	46 (29.6%)
Intention to quit ^d^	7.53 (2.17)	7.54 (2.30)	7.55 (2.1)	7.49 (2.06)

*Note.* Numbers may vary due to missing data.

^a^ low education = primary and basic vocational school, intermediate education = secondary vocational school and high school degree, high education = higher vocational school degree, college or university degree; ^b^ ACS = acute coronary syndrome, (non-)stemi = (non-) ST elevation myocardial infarction; ^c^ range from 0 = low nicotine dependence to 10 = high nicotine dependence; ^d^ range from 0 = weak intention to 10 = strong intention.

* *p*-value is significant at the 0.05 level.

### Interventions

#### *Standard in-hospital smoking cessation care (usual care)*

All patients received standard in-hospital treatment for smoking cessation which consisted of an assessment of their smoking behaviour and a personalised brief quit advice. Quit advice was largely provided by cardiologists (and occasionally by ward nurses) while patients were on the cardiac ward.

#### *Telephone counselling (TC) or face-to-face counselling (FC)*

There were two experimental conditions, one consisting of TC and one of FC. The two counselling methods started within one week after the inclusion of the patient and had a comparable structure and content. Nurses on cardiac wards followed the Ask-Advice-Refer strategy [69,70] prior to the counselling sessions. In accordance with this strategy, patients’ smoking behaviour was first assessed. Second, smoking patients were advised to quit, and third, they were referred to health professionals providing TC or FC outside the ward. Furthermore, nurses provided nicotine patches and information on their necessity and application to eligible patients. The counselling was provided by professional counsellors: in the TC, these were counsellors from the Dutch Expert Centre for Tobacco Control (STIVORO); in the FC, these were cardiac nurses trained in providing smoking cessation counselling. All counsellors received financial compensation. The main differences between the counselling methods were the delivery mode and intensity of the counselling. TC lasted for three months and consisted of seven telephone sessions of 10 to15 min (two hours in total). FC also lasted three months, consisted of six face-to-face sessions of 45 min and ended with a follow-up call eight weeks after the last session (4.75 h in total).

Existing protocols of TC and FC developed by the Dutch Expert Centre for Tobacco Control for the general smoking public were used, though adapted and fine-tuned to the situation of cardiac patients. The protocols focused on important determinants of smoking cessation and relapse such as self-efficacy and smoking-related attitudes [32,33,58]. The underlying theoretical methods of the two counselling methods were comparable and incorporated strategies based on the principles of motivational interviewing [38], enhancement of self-efficacy [71,72], goal-setting [73] and relapse prevention [74]. In contrast to TC, patients who were referred to the FC condition additionally received a workbook from their face-to-face coach. For each session the workbook contained the most relevant information and a few pieces of homework such as developing personalised strategies for dealing with high-risk situations or developing a personalised emergency plan for how to avoid absolute relapse when experiencing a lapse.

The design and main content of the TC and FC sessions are presented in Table 4. In seven successive counselling sessions, the counsellor assisted the patient to become smoke-free and/or maintain his or her smoking abstinence. The underlying theoretical model of the counselling sessions was the Transtheoretical Model by Prochaska and Diclemente [75]. This model posits that health behaviour change involves progress through five stages. The first three stages are pre-action stages (pre-contemplation, contemplation, preparation) where smokers have no or a weak intention to quit or prepare a quit attempt. The last two stages are post-action stages (action, maintenance) where smokers genuinely quit smoking and ideally maintain their abstinence. In line with each of these stages, the counsellor provided stage-matched information and applied strategies tailored to each individual patient in order to move forward from one stage to the next, to arrive finally at the smoking cessation maintenance stage.

**Table 4:** Main protocol of the Telephone Counselling (TC) and Face-to-face Counselling (FC) sessions for smoking cardiac patients

**Preparation**		**Action**		**Maintenance**		
Session 1 (Pre-) contemplation: Discussing the harmful effects of nicotine and the vicious cycle of nicotine dependence. Striking a balance between pros and cons of smoking and cessation.	Session 2 Preparation: Helping the patient to make the decision to quit smoking and prepare for quitting by providing information on nicotine withdrawal.	Session 3 Action: Providing information about the desire to smoke. Teaching strategies for dealing with craving and withdrawal symptoms.	Session 4 Action: Providing information about tempting (high risk) situations. Teaching coping strategies for tempting situations.	Session 5 Maintenance: Providing information about relapse and discussing strategies for how to prevent relapse to smoking. Developing an emergency plan in case the patient lapses.	Session 6 Maintenance: Choosing and discussing a theme relevant to the patient (i.e. stress, negative affect social pressure or controlling weight) to avoid relapse to smoking.	Session 7 Maintenance: Follow-up call. Evaluation of the cessation progress and discussing relapse prevention strategies.
All sessions • Boost patients’ motivation to quit smoking and/or maintain abstinence; • Adapt the content of the session to the particular situation and need of the patient; • Take into account that numerous patients quit at hospitalisation and retrospectively discuss pre-action stages to enhance consciousness of smoking cessation.						
TC: 15 min FC: 45 min week 1	TC: 15 min FC: 45 min week 2	TC: 15 min FC: 45 min week 3	TC: 15 min FC: 45 min week 4	TC: 15 min FC: 45 min week 5	TC: 15 min FC: 45 min week 7	TC: 15 min FC: 15 min week 12

Fine-tuning of TC and FC was directed at specific characteristics of cardiac patients previously shown to be impediments to smoking cessation (depression, anxiety, and Type D personality) [41,76-79], and targeted group-specific predictors of relapse [80-83]. A substantial number of the patients had not smoked since hospital admission. Some of them considered this temporary smoking restriction as a serious quit attempt, whereas others intended to resume smoking as soon as possible. To integrate these features as intervention components, protocols were adapted by the researchers including tips and tricks on the characteristics and particular situation of cardiac patients. The protocol contained information on how the content of the counselling sessions might vary and how it could be adjusted to the particular needs of the patient. An additional three-page information folder was developed and provided to cardiac patients in the intervention groups. This folder provided information and strategies on avoiding and dealing with risk situations for smoking where patients might experience negative affect and social inhibition, key characteristics of a Type D personality.

#### *Counselling combined with nicotine replacement therapy*

Reasons for the combination therapy of TC or FC with NRT in the study reported here were that antidepressants for smoking cessation (e.g. Bupropion) cannot be prescribed for all patients [84], NRT has been proven to be safe for cardiac patients [46,51] and equally effective as medical pharmacotherapy [31,84]. Cardiologists from the participating cardiac wards agreed on the prescription of transdermal nicotine patches for cardiac patients. The pharmacological therapy was therefore similar for all patients, essential to guarantee validity and enable conclusions to be drawn on the effectiveness of TC and FC. Owing to possible contra-indications in cardiac patients, cardiologists had to approve the delivery of nicotine patches for each individual patient. Eligible patients received NRT when indicated and when they smoked more than 10 cigarettes a day before hospital admission [85]. As treatment of eight weeks has been shown to be as efficacious as longer treatment periods [30], patients received the patches for eight weeks (at no cost).

#### *Recruitment and training of counsellors*

Professional telephone counsellors from the Dutch Expert Centre for Tobacco Control provided the TC (n = 4). The researchers provided additional training of four hours to the telephone counsellors. This training was necessary to inform the counsellors about specific patient characteristics. 

To recruit face-to-face counsellors from the participating hospitals two cardiac nurses per nursing unit were invited to become professional smoking cessation counsellors (n = 16). An expert team of senior trainers from the Dutch Expert Centre for Tobacco Control provided the training to the cardiac nurses. The training of face-to-face counsellors consisted of eight sessions of four hours spread over four days with a break of six weeks between the first and the last training sessions.

#### *Pilottesting of materials*

For a small number of patients (n = 6), face-to-face and telephone counsellors (n = 4) and cardiac nurses of the participating wards (n = 6), materials (questionnaires for patients, additional protocols for nurses and counsellors, workbook for patients, folder for patients) were pilot-tested by means of in-depth interviews. Even though the Dutch Expert Centre for Tobacco Control [58] had already evaluated their training for counsellors in general, the training was evaluated on a small scale, and focused on the counselling of cardiac patients in order to fine-tune the interventions for this target group. The pilot interview with cardiac patients and counsellors resulted in some amendments to the content and the adaptation of some of the materials in terms of sentence structure.

### Follow-up procedures

Six months after discharge (T1) patients were interviewed by telephone by members of a professional call agency to obtain follow-up data (see Figure 1). The 12-month follow-up measurements are still in progress (T2). The smoking cessation outcomes are assessed by means of validated reliable questions [86-88]. Telephone interviews are conducted in order to decrease the likelihood of attrition [10,89]. One postal reminder letter is sent 10 days before each of these telephone interviews. All patients who complete the 12-months interview are also invited to the hospital for biomedical tests of blood pressure, cholesterol and saliva.

Cardiac nurses are responsible for conducting the blood pressure tests, the cholesterol tests and the cotinine tests at the cardiac nursing ward. Cardiac nurses ask patients while conducting the blood pressure test if they have been smoking over the past seven days to assess point-prevalence abstinence. If patients claim not to have smoked any cigarettes, their saliva is tested on cotinine residuals by means of the NicAlert Cotinine Test Strip to test whether the self-reported smoking behavior is accurate. Results can be either positive (no cotinine detected) or negative (cotinine detected). Cardiac nurses register the results of the blood pressure, cholesterol and cotinine tests and send the findings to the research team.

### Measures

#### *Effectiveness study: outcome measures*

The primary outcome is seven-day point prevalence abstinence from smoking (PPA) after six and 12 months (0 = smoking;1 = non-smoking). PPA is the main outcome since it is considered to be the most sensitive and valid measure of smoking cessation [87]. Secondary outcomes are continued abstinence, quit attempts, and health outcomes. Health outcomes measured at T1 and T2 are new coronary events and hospital readmissions for coronary events. Cholesterol ratio and blood pressure are only assessed at T2 at the patients’ outpatient clinic. Intention to treat analyses will be applied [88] and smoking status is verified at T2 by a saliva sample. For these patients the outcome of the saliva test (detection of cotinine) will be taken as a control variable in the analyses to confirm the self-reported response of the smoking behaviour. This test has been proven to be a valid and reliable method for verification of smoking status [90].

#### *Cost-effectiveness study: Cost factors and resource use*

At T1 and T2, information on the other health economic outcomes is assessed for the past three months: visits to the general practitioner, cardiology outpatient visits, health related costs, rehospitalisations in the past six months, informal care costs, and health-related quality of life. Effects on productivity are measured in terms of absence from work owing to illness [91].

Resource use specifically for the interventions was recorded prospectively by the counsellors. This included counsellor time, use of materials, use of nicotine replacement patches and specialist time involved in cessation advice [92-94].

#### *Other measures*

A process evaluation was conducted at six-month follow-up in which patients were asked about usefulness of the interventions in total, their separate elements and patients’ adherence to NRT and counselling sessions. The baseline measurement and both post-tests also assess factors that are related to smoking cessation and/or relapse among this patient population. These include demographics; smoking-related factors [95]; disease-related factors; psychological states including depression and anxiety [96], Type D personality [97]; smoking cessation-related cognitions and motivation to quit [10,98]. To measure smoking-related cognitions and motivation to quit, existing questionnaires are used [86,99] which are based on previous social-cognitive models [75,100,101]. To assess the expected differential effects of the interventions for SES, SES is assessed in terms of education level and income [86].

### Statistical analyses

Data is analyzed with SPSS. Descriptive statistics are used to describe the sample, and to compare the groups at baseline, ANOVA analyses and chi-square analyses are applied. In order to determine possible selective loss at follow-up, attrition analyses are conducted. The effects of the interventions on smoking cessation outcomes and possible interaction effects (condition * SES/condition * intention to quit) are tested by means of logistic regression analyses. The hospital group size of eight is inadequate for reliably testing the existence of variation by a multi-level model [102]. Therefore, a fixed-effect regression approach is likely to be used to eliminate possible hospital-level effects. Baseline variables are included as covariates to correct for possible baseline differences, to reduce unexplained variance and to increase the power of the tests. To analyse effects on rehospitalisation, new cardiac events, number of visits to the cardiologist and cardiovascular-related death, multiple regression analyses are used. Effects of the interventions on blood pressure, TC/HDL cholesterol, and intentions to quit are tested by means of repeated measures analyses of covariance. Logistic regression analyses are also used to examine predictors of smoking cessation overall and within subgroups. Patients in the experimental groups are compared by paired sample t-tests for level of appreciation of the intervention. Economic evaluation analyses are performed by applying specific cost-effectiveness calculation formulas. Moreover, a health-economic Markov Model is built to estimate long-term costs and health effects.

## Discussion

Two different interventions were designed that combined intensive smoking cessation counselling – either delivered face-to-face or by telephone – and NRT. The interventions were designed to overcome the weaknesses of usual care and minimal interventions being too brief and not including recommended elements of smoking cessation treatment guidelines [30]. To overcome a second drawback of current interventions, i.e. the implementation and feasibility difficulties reported by ward nurses in terms of providing brief support on smoking cessation, the counselling (TC and FC) was delegated to smoking cessation professionals tasked with formal responsibility who had enough time to spare. As nurses applied the Ask-Advice-Refer approach, attention was paid to patients’ smoking behaviour at hospital admission while being referred to external professionals. This approach was assumed to be feasible in busy nursing units where nurses lack time and skills to provide smoking cessation support themselves [69,70]. Offering smoking cessation treatment as an adjunct to hospital-initiated programmes shortly after hospital discharge lastly overcame the limitation of cardiac rehabilitation programmes which usually do not start within the first four weeks after discharge and only pay very brief attention to smoking cessation.

### Study strengths

This study is a multi-centre trial carried out in eight hospital cardiac wards from different geographical regions of the Netherlands. Most randomised controlled trials on preventative interventions for smoking cardiac patients are conducted in only one or a few hospitals, so the information gathered from eight hospital wards improves generalisation of the study results. Cross-over randomisation was conducted at the ward level by sequentially implementing the conditions, which was done to overcome problems related to traditional randomised controlled trials. This design meant contamination between patients was avoided, as well as learning effects on ward nurses. The chosen design also circumvented nurses’ mistakes in performing the interventions. Moreover, the experimental design allowed us to control for confounding effects such as seasonal influences or other extraneous events. Last but not least, a cost-effectiveness study was carried out alongside the standard clinical trial, which allows us to estimate the additional costs and benefits of the interventions compared with a control group receiving usual care.

### Methodological considerations

Although we made efforts to include all eligible smoking cardiac patients within the a time frame of one year and to register data on those eligible patients not enrolled in the study, the baseline results suggested that neither task was performed as intended. Nurses were asked to register the refusals of patients not enrolled in the study but this was not well done. There are indications that in some hospitals there were more refusals than in others. The inclusion period was expanded to 18 months as the inclusion of smoking cardiac patients in hospital cardiac wards took longer than expected. Low inclusion rates were related to ward factors such as insufficient patient admissions, few patients who met the inclusion criteria of the research, and limited admission capacities of cardiac wards. Other factors hindering patient inclusion in the study were high workloads on cardiac wards, non-collaborative nursing teams, and little interest in smoking cessation programmes at the general hospital. These factors have previously been shown to impede implementation of smoking cessation interventions in cardiac wards [103-105]. The inclusion rate in the FC group was relatively low which might be related to its time-consuming treatment form, resulting in a skewed distribution of patients with 157 patients in the FC group, 223 in the TC group and 245 in the UC group. Moreover, inclusion rates varied considerably between cardiac wards and this suggests the likelihood of non-representative outcomes of the effectiveness study for those cardiac wards that only included a few patients per group. We also note that there were differences in nicotine dependence between the experimental groups and the usual care group, as nicotine dependence was significantly higher in the experimental groups. Altogether these considerations pose the limitation of a selective sample of cardiac patients, implying the potentially limited generalisability of the study results.

## Conclusion

Feasible evidence-based smoking cessation intervention approaches are required to support cardiac patients in quitting smoking in order to reduce future risks of cardiac events. Since subgroups may profit more from a different type of intervention and should receive the most (cost-)effective one, this study compares two intensive counselling methods - one provided face-to-face and the other by telephone - to each other and to usual care on their costs and effects on smoking abstinence. Although the inclusion period was expanded and not all eligible patients could be included, the results of this multi-centre trial will be able to provide valuable insight into the (cost-)effectiveness of both counselling interventions.

## Abbreviations

SES, Socio-economic status; CMIS, Minimal intervention strategy for cardiac patients; NRT, Nicotine replacement therapy; Type D, Distressed personality type; FC, Face-to-face counselling; TC, Telephone counselling; ICD-10, International Classification of Diseases; STIVORO, Dutch Expert Centre for Tobacco Control; PPA, Seven-day point prevalence abstinence from smoking; TC/HDL cholesterol, Total/high density lipoprotein cholesterol ratio.

## Competing interests

All authors declare that they have no competing interests.

## Authors’ contributions

NB carried out the study and had a major role in data collection, data analysis and data interpretation. She also drafted the manuscript. CB conceived of the study, design, coordination and helped to draft the manuscript. LL, AM, FV and HdV revised the manuscript critically for important intellectual content. All authors read and approved the final version of the manuscript to be published.

## Authors’ information

NB is the primary PhD investigator of the current study on the (cost-)effectiveness of two intensive counselling methods for smoking cessation in persons with coronary heart disease. CB is associate professor in health psychology and received her PhD for studies on a minimal smoking cessation intervention strategy for cardiac inpatients. LL is professor in health psychology and has great expertise in and scientific knowledge on developing and evaluating behavioural interventions in general. AM is assistant professor in health psychology and has been involved in many projects on smoking cessation as co-promoter or advisor. FV is cardiologist and has been chair of Dutch Society of Cardiology. HdV is professor in health communication and has done a lot of research on smoking prevention and cessation.

## Pre-publication history

The pre-publication history for this paper can be accessed here:

http://www.biomedcentral.com/1471-2261/12/33/prepub
